# Langmuir Monolayer Fiber Drawing of Aligned Copper Nanowires

**DOI:** 10.1021/acs.langmuir.6c03471

**Published:** 2026-09-30

**Authors:** Chih-Chun Hu, Chingping Chang, Zi-Tao Chu, Tzu-Shan Kao, Yu-Jou Lin, Yi-Chen Wang, Rohit Dagar, Kevin Chiou

**Affiliations:** Department of Materials and Optoelectronic Sciences, 34874 National Sun Yat-Sen University, No. 70, Lianhai Road, Kaohsiung 80424, Taiwan

## Abstract

One-dimensional (1D) nanomaterials are valued for their anisotropic morphology, but they have tendencies to aggregate uncontrollably due to intermolecular interactions and entanglement. We developed a method that can align and bundle 1D nanomaterials into yarn-like microfibers with copper nanowires (CuNWs) as an example of applicable 1D nanomaterials. The method begins by spreading CuNWs into a Langmuir monolayer. The CuNWs form a cohesive, entangled network over the subphase surface, so the CuNWs can be drawn up from one point to collapse the Langmuir monolayer into a microfiber. The drawing motion aligns the CuNWs by pulling them against the subphase’s surface tension. During drawing, residual solvent in the microfiber evaporates to compress CuNWs and facilitate conformal contacts. The assembled microfibers are held together by van der Waals interactions and entanglement between misaligned components. The CuNW microfibers have a Herman’s orientation factor of 0.84, representing significant alignment. These microfibers are cohesive enough to be manipulated into knots and twists. The CuNW microfibers were also examined for their stress–strain properties and electronic conductivity. The discovered technique does not rely on designed surface properties, so 1D nanomaterials with sufficient aspect ratios have a chance to be aligned using this method, including silver nanowires, polystyrene fibers, zinc oxide nanorods, and mixtures of 1D nanomaterials. From its flexible processability, we believe the discovered technique would complement various modifications, such as surface modifications or application-specific chemical compositions.

## Graphical abstract

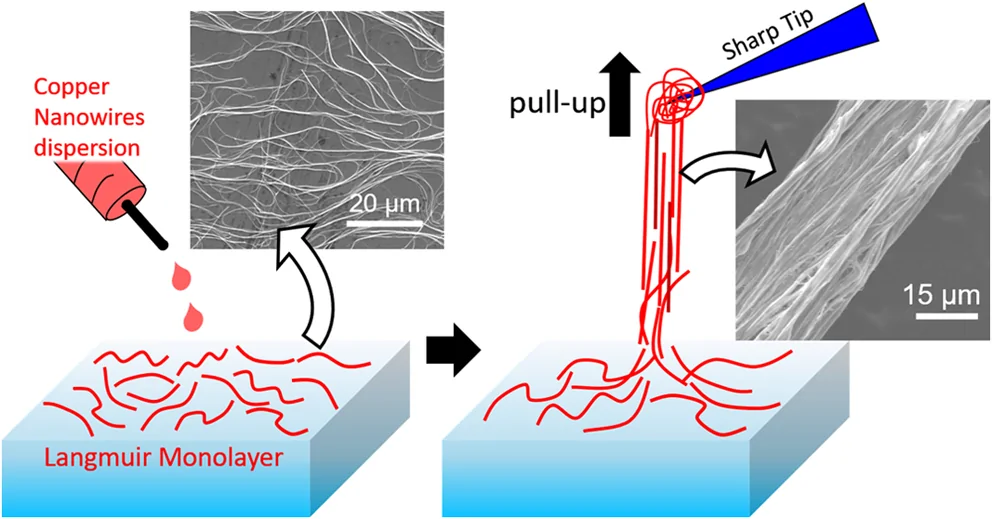

## Introduction

Nanomaterials’ assembly has a prominent role in the discovery and usage of nanomaterials. In particular, alignment of one-dimensional (1D) nanomaterials contributes to many applications such as guided cell growth,^1–3^ field effect transistors,^4–6^ optoelectronic components,^7–9^ and high-performance mechanical components.^10,11^ Orienting 1D nanomaterials in one direction directs their anisotropic properties. A mass-producible 1D nanomaterial is the copper nanowires (CuNWs), which can form anisotropic structures with a high degree of alignment. CuNWs have high aspect ratio, electrical conductivity, and thermal conductivity, so they can find applications as conductive pathways,^12–15^ thermal dissipators,^16–18^ conductive meshes,^19–23^ and high-surface-area catalysts.^24–26^ Alignment and assembly process for CuNW can further benefit various applications, and the described process would be applicable to other 1D nanomaterials as well.

Many alignment strategies of 1D nanomaterials involve additives or surface modifications, but these additions could alter the properties of 1D nanomaterials. One assembly methodology is self-assembly, which relies on engineering interactions between adjacent particles to interact and assemble into the desired structures.^27–29^ Self-assembly techniques were used to bundle 1D nanomaterials such as carbon nanotube (CNT),^30^ silicon nanowires,^31^ and other metallic nanowires.^32^ Yet, modifying surfaces and the use of surfactants add unwanted impurities and surface defects. Another alignment methodology is to use external forces to assemble 1D nanomaterials into aligned structures.^33–35^ Extruding 1D nanomaterials with a processing aid generates a shear force that aligns the 1D nanomaterial in the extrusion direction.^6,36^ However, the shear force on 1D nanomaterial mixtures can cause unwanted damage. Another method applies external fields onto Langmuir monolayers, such as electrostatic fields, magnetic fields, or mechanical agitation, to align the 1D nanomaterials in the desired direction.^37^ Aligned nanomaterials of the Langmuir monolayer could transfer to a substrate, but this limits the aligned 1D nanomaterials to be dependent on the substrate.^38,39^ Some alignment methods are specific to the 1D nanomaterial, such as 1D nanomaterial growth and CNT fiber drawing. Some 1D nanomaterials can be grown as unidirectional arrays,^40–42^ but finding the growth parameters can be challenging. Dense CNT arrays can be drawn directly into fibers of aligned CNTs.^43–45^ Yet, CNT arrays can be challenging to synthesize in bulk. Flexible 1D nanomaterials, such as polymer nanofibers, can be electrospun directly into mats of aligned nanofibers. Yet, alignment via electrospinning requires specifically designed rotating substrates, such as a drum, a cage, or a cone.^46–49^ While there are options for 1D nanomaterials’ alignment and fabrication into yarn-like fibers, there is no technique that can turn a wide range of 1D nanomaterials into free-standing aligned fibers without introducing impurities or damaging the 1D nanomaterials.

A suitable alternative alignment method for 1D nanomaterials should utilize the materials’ shared high aspect ratio to generate yarn-like bundles. At sufficiently high aspect ratios, 1D nanomaterials will appear more flexible compared to their bulk forms.^50,51^ When 1D nanomaterials are dispersed over a liquid surface as Langmuir monolayers, these 1D nanomaterials are trapped in a two-dimensional (2D) surface, and they have limited degrees of freedom. The trapped 1D nanomaterials can entangle by curling around each other. Entangled 1D nanomaterials form a cohesive monolayer that can be drawn up from a single point to be collapsed into a continuous microfiber. The described technique is tentatively called Langmuir monolayer fiber drawing (LMFD). The process is unlike the Langmuir–Blodgett film deposition technique or the Langmuir–Schaffer deposition technique because it bundles 1D nanomaterials into microfibers without a substrate. Instead, LMFD relies on the 1D nanomaterials’ high aspect ratio to have functional flexibility and entanglement, so they can form free-standing yarn-like microfibers.

LMFD requires materials that can form Langmuir monolayers. Some materials are more suitable because they can form Langmuir monolayers after synthesis without further processing. Additionally, 1D nanomaterials must have a sufficient aspect ratio to have functional flexibility.^50,51^ CuNWs can form Langmuir monolayers right after synthesis and have an aspect ratio of 1000. With an aspect ratio of 1000, these CuNWs behave like flexible strands that readily curl and entangle. The foreseeable limitations of LMFD are different from those of other assembly techniques, so LMFD can be an alternative technique for bundling of 1D nanomaterials into free-standing microfibers.

This report of LMFD focuses on CuNWs due to ease of production and functionality. In a typical LMFD process, dispersions of CuNWs are spread across an ethanol subphase. While Langmuir monolayers typically use water subphases, ethanol was chosen as the subphase for LMFD because ethanol’s surface tension of 22.1 mN/m is lower than water’s 72.8 mN/m. High surface tension helps spread material across the subphase surface, but water surface tension pulls on a microfiber too strongly during LMFD. Ethanol has an intermediate surface tension that can spread nanomaterials and allow LMFD without unwanted fracture. The Langmuir monolayer at a high enough concentration, indicated by surface pressure, is lifted up by a 27 gauge hypodermic needle from one point to collapse it into a microfiber. During LMFD, residual ethanol evaporates to compress and bundle CuNWs together. Other types of 1D nanomaterials were also tested with LMFD, and they could also be fabricated into microfibers. These results suggest that LMFD can be a suitable alternative for bundling 1D nanomaterials into yarn-like microfibers.

## Results and Discussions

The Langmuir monolayer fiber drawing method (LMFD) of CuNW is an assembly process that turns dispersed 1D nanomaterials into a yarn-like bundle of aligned 1D nanomaterials. LMFD can be divided into two steps to turn dispersed CuNWs into a microfiber (Figure 1a). The first step is to trap CuNW on a liquid surface to partially limit the movement of CuNWs to the 2D plane. The monolayer of CuNWs could then be drawn up from one point, form a funnel-like structure, and collapse into a single microfiber of CuNWs. These two steps limit the orientations of CuNWs to better control CuNW interactions and form bundles. During the LMFD process, there is a visible funnel that draws in surrounding CuNWs into a microfiber (Figure 1b). During the LMFD process, a clean silicon wafer can be used to scoop up and lift out the funnel-like shape. The funnel will dry and collapse onto the substrate, which fixes the CuNWs in place for further observation. Under the scanning electron microscopy (SEM), the pyramidal structure appears flattened, and the CuNWs are shown to align and gather into a microfiber (Figure 1c). CuNWs’ concentration around the funnel appears to be much higher than other spots of the Langmuir monolayer because the CuNWs are being pulled into the funnel from all sides. The obtain microfiber appears to be bundled CuNWs aligned in the general direction of the fiber. The CuNWs are aligned to the microfiber being pulled in two opposing directions when the microfiber is being drawn. The Langmuir monolayer is being pulled up from the subphase surface. At the same time, surface tension from the subphase pulls down upon the funnel-like structure to resist the microfiber from being pulled up. The combined interactions create tension in the longitudinal direction of the microfiber and align the constituent CuNWs (Figure 1d). During fiber drawing, the CuNWs are held together via van der Waals interactions, entanglement, and residual solvent from the subphase. When residual solvent evaporates, the microfiber consolidates the aligned structure to yield the yarn-like bundle of aligned CuNWs.

**Figure 1:**
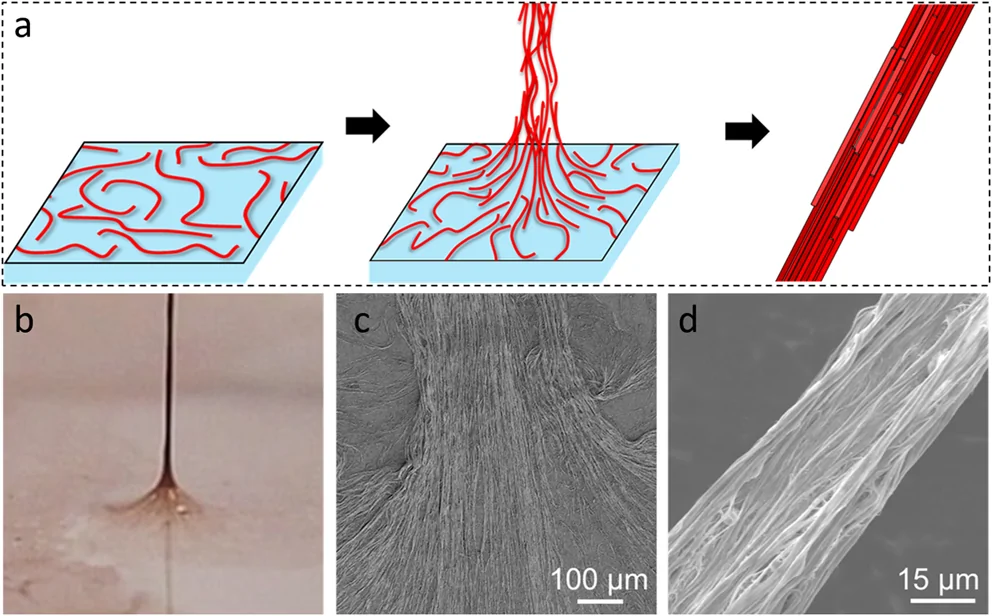
Langmuir monolayer of 1D nanomaterial drawn up into a microfiber shown as a schematic (a). Experimental picture of the CuNW monolayer drawn up into a microfiber (b). SEM image of a funnel-like intersection between the microfiber of CuNW and the connected Langmuir monolayer (c). SEM image of the drawn CuNW microfiber (d).

LMFD aligns CuNWs by relying on the fiber drawing motion. The microfibers are held together by van der Waals interactions and entanglement between adjacent CuNWs. Drop-cast CuNWs show high aspect ratios that impart good apparent flexibility to facilitate entanglement between CuNWs (Figure 2a). By averaging 20 strands of CuNWs using the SEM, we found that the average lengths of the CuNWs were 41.1 ± 7.7 μm long. Using transmission electron microscopy (TEM), we were able to find that the CuNWs are 18.6 ± 3.5 nm wide (Figure 2b). SEM images can see that strands of CuNWs appear to be much longer than the average, but these are aggregates of CuNWs extending beyond the lengths of individual CuNWs. The X-ray diffraction (XRD) spectrum shows that CuNWs consist only of copper when compared to JCPDS 04-0836, showing characteristic (111), (200), and (220) peaks (Figure 2c). Semicrystalline CuNWs of Figure 2a have significantly more flexibility compared to bulk copper, so their apparent flexibility can be attributed to their elongated morphology. Fourier transform infrared (FT-IR) shows that the surface of CuNWs is devoid of oleylamine’s strongest IR bands at 2922, 2853, 1466, and 796 cm^–1^ (Figure 2d). The lack of oleylamine suggests that CuNWs’ surfaces must consist of copper or its oxidized derivatives. Combining the characterization information, these CuNWs do not have other surface groups that can interact. Metallic copper cannot facilitate dipole–dipole interactions due to the lack of a permanent dipole. Among the most common intermolecular interactions, the remaining van der Waals interactions become the most likely types of interactions between these CuNWs. The conditions of Langmuir monolayer formation cannot avoid the oxidation of CuNW surfaces, but the thin oxide layers that form over CuNW surfaces would not contribute to the interaction between adjacent CuNWs. Other nanomaterials have been reported to rely on van der Waals interactions, such as graphite, hexagonal boron nitride, and transition-metal dichalcogenides.^52,53^ Hence, it is plausible for LMFD drawn bundles of CuNWs to be held together in a similar manner as other van der Waals solids. Significant intermolecular interactions between adjacent CuNWs help the microfiber remain cohesive during LMFD and resist the subphase’s surface tension, pulling it down. Using Fiji’s plugin OrientationJ, CuNW microfiber and a noncompressed CuNW Langmuir–Blodgett film were compared via color-survey images^54^ (Figure 2e). CuNWs in the microfiber are the most colored in the same blue, indicating a similar orientation as opposed to the CuNW Langmuir–Blodgett film’s mix of colors corresponding to different orientations. The Fourier transform of the SEM diagram shows a 90° line perpendicular to the microfiber’s longitudinal direction, which indicates that CuNWs are aligned along the longitudinal direction (Figure 2e). Orientation distribution curves could be generated from the SEM images, which show that the microfiber of Figure 2e is mostly aligned in the 0° direction. The CuNW microfiber has a Herman’s orientation factor of 0.84 compared to the CuNW Langmuir–Blodgett film’s factor of 0.54. The CuNW microfiber’s Herman’s factor of 0.84 corresponds to significant alignment and the presence of some defects in the microfiber. The CuNW Langmuir–Blodgett film’s Herman factor of 0.54 suggests that there is some directionality, but the film is close to isotropic.

**Figure 2:**
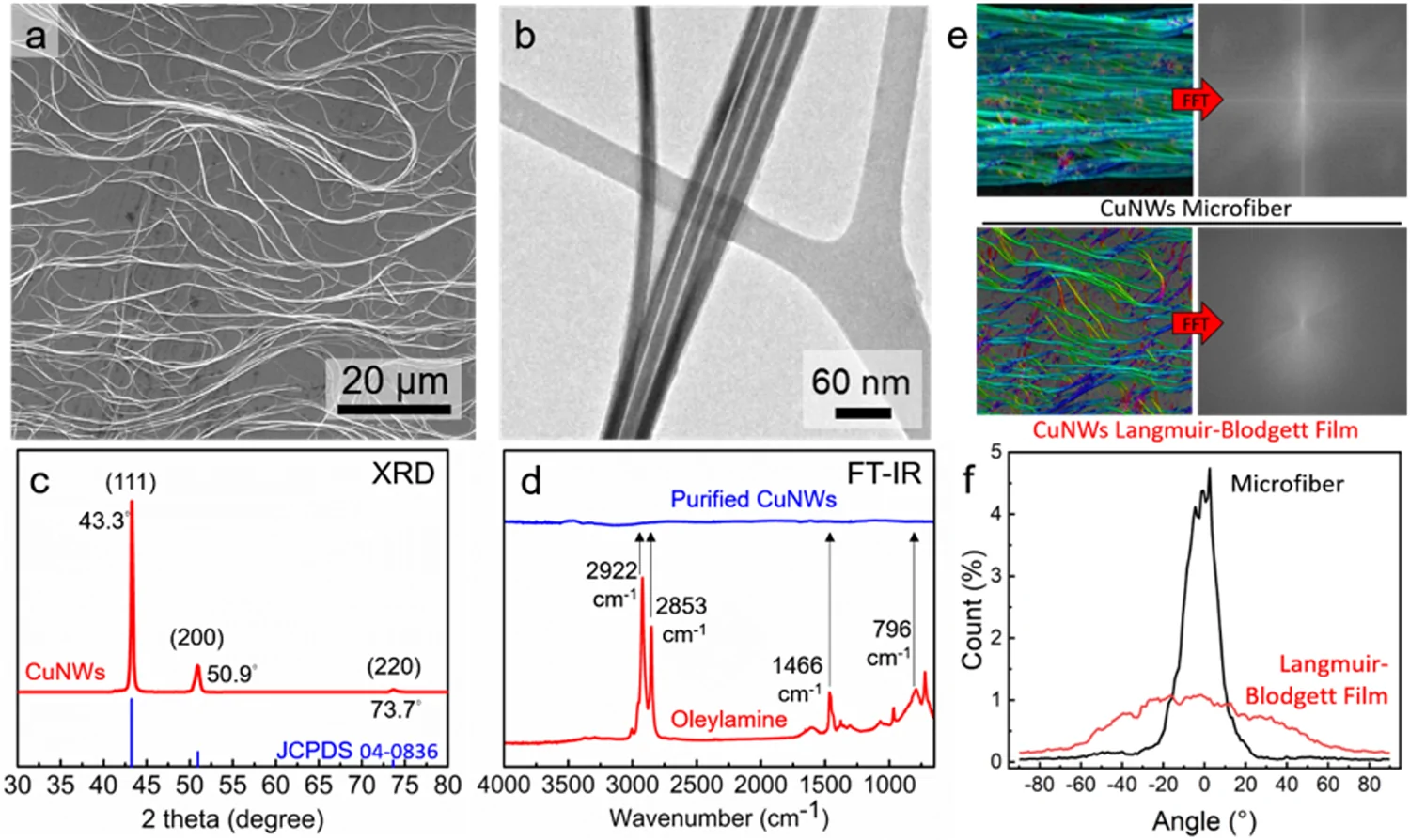
CuNWs were synthesized and characterized by SEM (a), TEM (b), and XRD (c). Using FT-IR, purified CuNWs were compared to oleylamine, which was used as a surfactant during CuNW synthesis (d). Fiji with OrientationJ was used to evaluate the CuNWs’ orientation as color surveys, fast Fourier transform (FFT) diagram (e), and distribution curves of a CuNW microfiber and a CuNW Langmuir–Blodgett film (f).

LMFD uses van der Waals interactions to assemble and bundle CuNWs into microfibers, and the van der Waals interactions’ commonality helps LMFD to be applicable to various 1D nanomaterials. In addition to CuNWs, other 1D nanomaterials were also processed via LMFD (Figure 3). Silver nanowires synthesized from hydrothermal reaction can be drawn into microfibers because they have high-aspect-ratio morphologies similar to CuNWs (Figure 3a). Polystyrene (PS) electrospun fibers with an average diameter of 3.5 μm are much wider than the CuNWs or silver nanowires, and these PS fibers were also able to be assembled via LMFD (Figure 3b). Despite the larger diameters, PS fibers’ long length imparts the polymer fibers with high aspect ratios similar to CuNWs, such that LMFD can be applied. A mixture of CuNWs and zinc oxide rods was also assembled via LMFD into a yarn-like microfiber with bundled CuNWs and zinc oxide nanorods aligned in the same direction (Figure 3c). Another microfiber was drawn from a mixture of thin CuNWs and thick PS fibers, which shows that fibers of mixed sizes can align in the same direction when processed via LMFD (Figure 3d). The four types of microfibers consist of different materials, and they all have averaged aspect ratio above 1000. When 1D nanomaterials with an averaged aspect ratio less than 500, LMFD cannot reliably be applied to draw aligned microfibers. The lower minimum exists because 1D nanomaterials with aspect ratios lower than 500 do not entangle enough during the LMFD process. When a 1D nanomaterial’s aspect ratio is between 1000 and 500, the applicability of LMFD is difficult to predict due to differences in the intrinsic flexibility of each 1D nanomaterial. While more extensive testing is necessary, results thus far show that LMFD is applicable for a wide range of 1D nanomaterials with sufficient aspect ratios to entangle and form conformal contacts.

**Figure 3:**
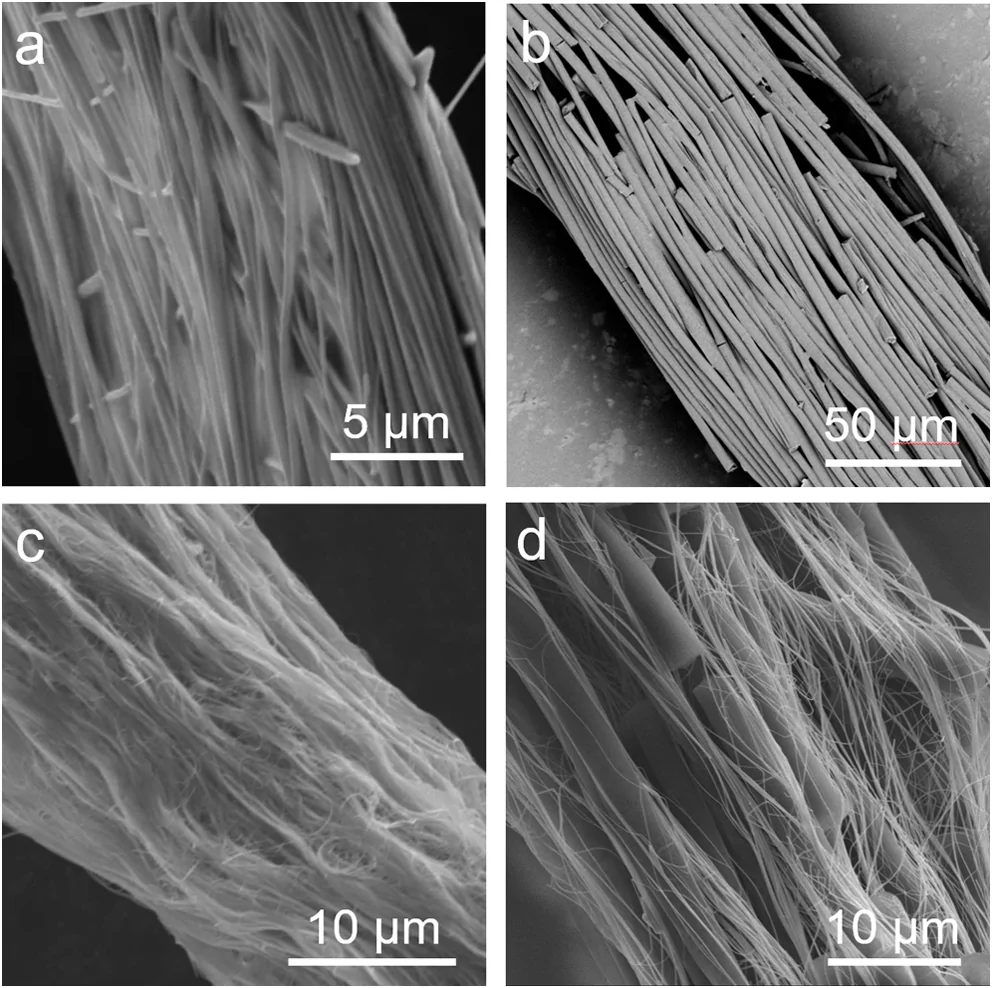
Using the LMFD method, microfibers can be drawn from silver nanowires (a), PS nanofibers (b), mixtures of CuNWs and zinc oxide nanorods (c), and CuNWs and PS nanofibers (d).

LMFD is derived from the Langmuir monolayers, so the properties of the Langmuir monolayer will affect the microfibers. When a microfiber is drawn, a funnel-like structure collapses the Langmuir monolayer into a microfiber. The diameter of the drawn microfiber is directly related to the areal concentration of the 1D nanomaterials in the Langmuir monolayer. The amount of 1D nanomaterials in a Langmuir monolayer can be described with its surface pressure measured using a Wilhelmy plate.^55,56^ The LMFD of CuNWs from Langmuir monolayers with increasing surface pressure yields CuNW microfibers of increasing diameters up to 4.0 mN/m (Figure 4a). Increasing the surface pressure beyond 4.0 mN/m would instead lead to a decreasing diameter of the microfibers. This transition can be associated with the increased entanglement between CuNWs to cause the Langmuir monolayer to change from a fluid-like phase to a more static solid-like phase.^57,58^ The transition in the CuNW Langmuir monolayer is marked by the sharp increase in surface pressure after 4.0 mN/m (Figure 4b). The transition also coincides with the onset of a decrease in microfiber diameter starting at 4.0 mN/m (Figure 4c).

**Figure 4:**
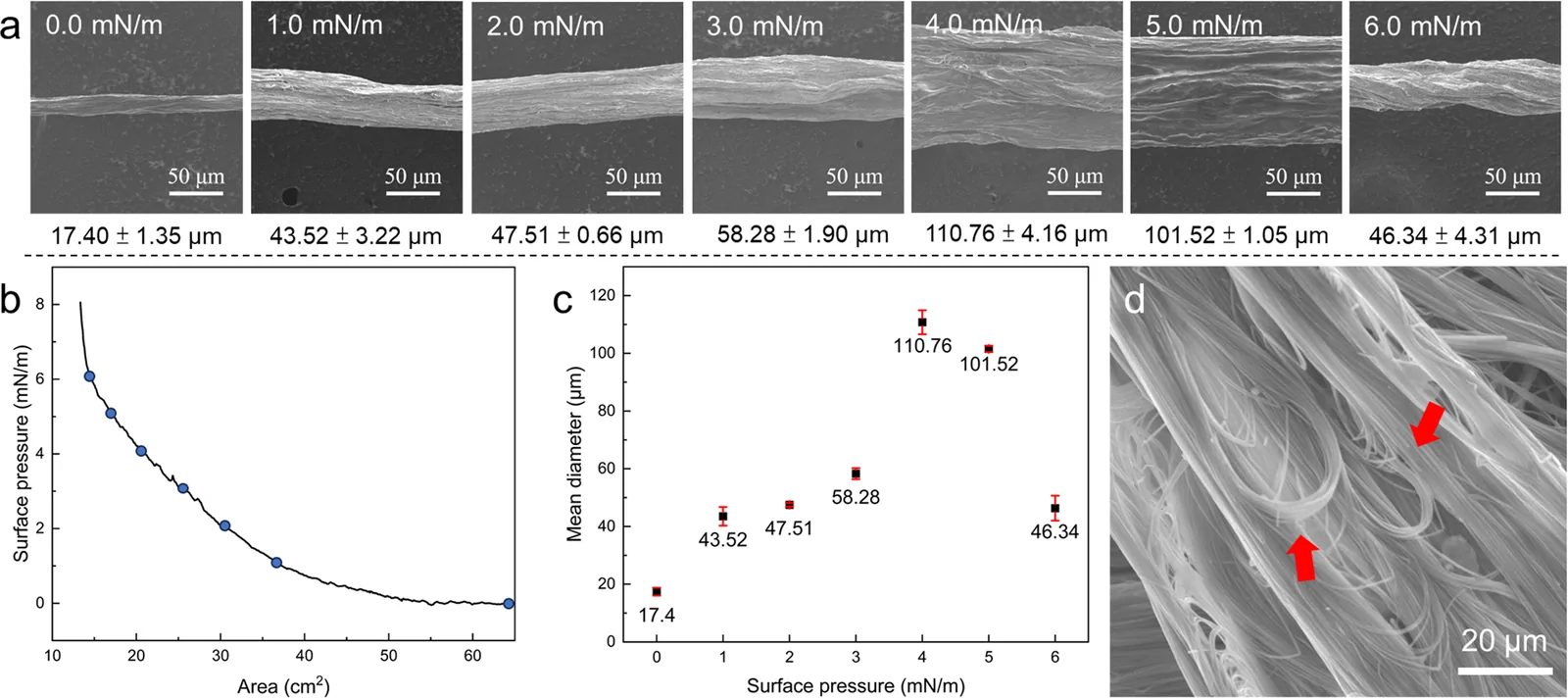
SEM images of CuNW microfibers of different diameters drawn from different surface pressures (a). Surface pressure recorded by a Wilhelmy plate and controlled by the surface area of the Langmuir monolayer (b). Diameters of CuNW microfibers at different surface pressures (c). SEM image of a CuNW microfiber’s surface showing the CuNW compressed into tight turns (d).

Microfibers drawn from liquid-like Langmuir monolayers have the right amount of interactions to hold adjacent CuNWs together and sufficient free volume to rearrange into alignment. Yet, sharp acute turns can be observed in the constituent CuNWs because the CuNWs are pressed together during fiber drawing (Figure 4d). During LMFD, residual solvent starts to evaporate immediately after leaving the subphase surface, which exerts a radial force to compress the constituent nanomaterials together. Some CuNWs may adjust their alignment with the microfiber direction. CuNWs with larger deviations that cannot rearrange completely will instead fold to generate the sharp acute turns. Microfibers drawn from liquid-like phase Langmuir monolayers have suitable mobility to rearrange and remain cohesive. If the Langmuir monolayer is in a solid-like phase, the CuNWs with large deviations would not rearrange, and they cannot form a long microfiber. The compression is mainly from evaporating residual solvent and the tension in the LMFD process, and the combined forces bundle 1D nanomaterials into microfibers.

In addition to arranging 1D nanomaterials into the desired morphology, LMFD can generate bulk structures that are large enough and sufficiently stable to handle. A fiber drawn via LMFD from a large-area Langmuir monolayer can be as long as 13 cm (Figure 5a). Currently, LMFD is done by hand, which introduces instabilities that affect fiber quality. If the LMFD process can be automated, even longer and more uniform microfibers may be obtained. The CuNW yarn-like microfibers from LMFD are stable enough to be handled and manipulated using tweezers (Figure 5b). The microfibers can be tied together and observed under SEM (Figure 5c). The CuNWs in the microfiber remain oriented in the longitudinal direction of the microfiber, and they do not show fraying from the physical manipulation. The CuNW microfibers could also be twisted into spirals without significant fraying of CuNW (Figure 5d). Further twisting the microfiber will cause coils to form in the microfiber, and some CuNW will fray away due to the increase in curvature and mechanical distortion (Figure 5e). The capability for microfibers to stay in one piece upon mechanical manipulation demonstrates the cohesion of the material. Mechanical properties of a CuNW microfiber were evaluated from a stress–strain curve. The CuNW microfibers have an average Young’s modulus of 255.7 ± 50.1 MPa, a tensile strength of 0.942 ± 0.297 MPa, and a fracture strain of 0.227 ± 0.074% (Figure 5f). For CuNW microfibers that are held together by van der Waals interactions and entanglement, the microfibers are expected to appear soft as the individual nanowires slide against each other during deformation. Electrical conductivity of the CuNW microfibers was measured using a 4-point probe. Immediately after preparation, a CuNW microfiber has a conductivity of 1.26 × 10^5^ S/m (Figure 5g). However, the bare CuNWs quickly oxidize under ambient conditions, and the microfiber’s conductivity decreases rapidly within 3 h. While additional testing and examination on the properties of the microfibers are necessary, we suspect they would exhibit similar trends as other bundled 1D nanomaterials. With meticulous design, LMFD may produce microfiber components for microscopic applications that demand directionality in its nanomaterial components, such as field effect transistors, microrobotic actuators, and waveguides.

**Figure 5:**
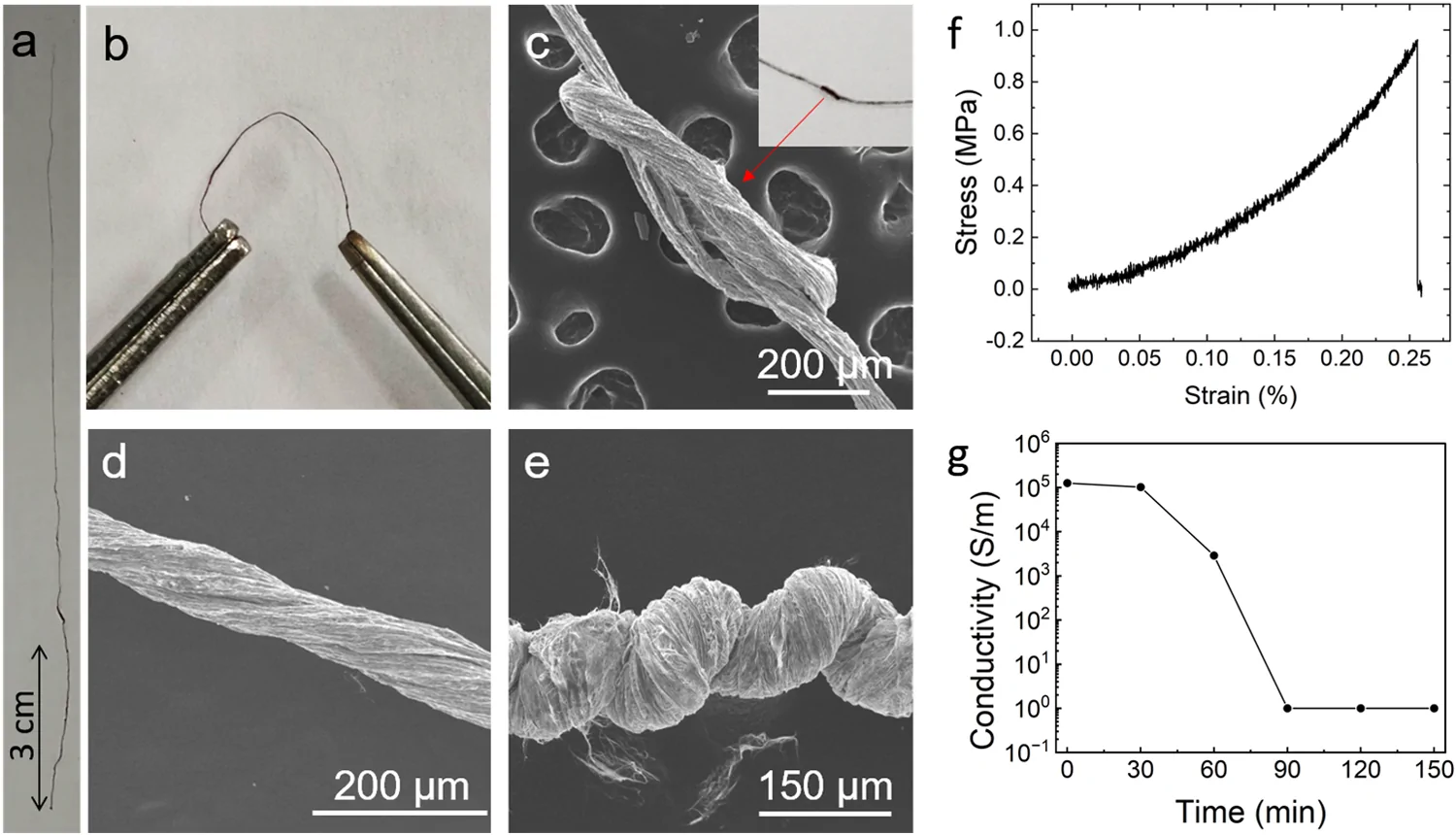
CuNW microfiber using LMFD with a length of 13 cm (a). Tweezers can be used to pick up and manipulate the fibers (b). SEM images of knots tied with the microfiber (c). Microfibers could also be lightly twisted (d) or overtwisted to form coils (e). Stress–strain curve of a straight CuNW microfiber (f). Electrical conductivity of the CuNW microfiber was measured over time (g).

## Conclusions

Aligning and bundling of 1D nanomaterials into microfibers are challenging operations that can be completed using LMFD. Many 1D nanomaterials are synthesized as randomly oriented powders, and these 1D nanomaterials may not have suitable methods for subsequent alignment and applications. LMFD provides an alternative method for assembling dispersible 1D nanomaterials by relying on the 1D nanomaterials’ high aspect ratio and strand-like morphology. LMFD starts by forming a Langmuir monolayer from a dispersion of the 1D nanomaterial. The Langmuir monolayers could then be drawn upward from a single point, which generates a funnel-like structure that simultaneously collects, aligns, and bundles the 1D nanomaterials into a microfiber. The LMFD technique can be applied to various 1D nanomaterials, such as silver nanowires, PS fibers, and mixtures of different 1D nanomaterials. Because LMFD relies on van der Waals interactions and entanglement that are common among 1D nanomaterials, LMFD may align and bundle a wide range of 1D nanomaterials with sufficiently high aspect ratios. The obtained microfibers also showed good cohesion to be twisted and tied without the 1D nanomaterial bundles falling apart. With its versatile processing capabilities, LMFD can help generate 1D nanomaterial structures with applications in microrobotics, artificial actuators, field effect transistors, and other applications that benefit from aligned 1D nanomaterials.

## Materials and Methods

### Materials

Copper­(II) chloride dihydrate (99%, abbreviated as CuCl_2_·2H_2_O), oleylamine (80–90%, abbreviated as OAm), and oleic acid (99%, abbreviated as OAc) were purchased from Acros Organics. Glucose (99%), Triton X-100, PS (MW 192,000), poly­(vinylpyrrolidone) (*M*_w_ 55,000, abbreviated as PVP), sodium dodecyl sulfate (>99%, abbreviated as SDS), and silver nitrate (99.0%, abbreviated as AgNO_3_) were purchased from Sigma-Aldrich. Sodium chloride (99.5%, abbreviated as NaCl), zinc chloride (99%, abbreviated as ZnCl_2_), and sodium carbonate (99.8%, abbreviated as Na_2_CO_3_) were purchased from Showa Chemicals Company.

Solvents such as acetone (98%), ethanol (190 proof), and hexane (98%) were purchased from Sigma-Aldrich. Isopropanol (98%) was purchased from Tedia Inc. Chloroform (98%) was purchased from Honeywell Fluka. Dimethylformamide (98%, abbreviated as DMF) was produced by Duksan Chemicals. Ethylene glycol (99%, abbreviated as EG) was produced by J. T. Baker Chemical Company.

### Synthesis of Copper Nanowires

Synthesis of CuNWs followed a method previously reported by Li et al.,^59^ and purification procedures were modified from a prior publication by Wang et al.^60^ A typical synthesis of CuNWs began by dissolving 33.5 mg of CuCl_2_·2H_2_O and 49.5 mg of glucose into 5.0 mL of deionized water (abbreviated as DI water). In a separate solution, 5.0 μL of OAc and 0.50 mL of OAm were dissolved in 0.875 mL of ethanol. The two solutions were combined and held at 50 °C and stirred for 12 h. After the aqueous mixture reacted for 12 h, it was transferred to a hydrothermal reactor to react at 120 °C for 6 h. After the hydrothermal reaction, the mixture was cooled to room temperature before collecting a red-brown mixture of dispersed CuNW. Purification of the mixture began by transferring the red-brown mixture to a 15 mL Falcon tube, followed by the addition of 3.0 mL of hexane. The combined mixture was shaken by hand for 5 min and left to phase separate on its own. CuNWs gathered at the interface between hexane and water, so they could be collected and dispersed into hexane for storage.

To ensure purity, XRD was used to examine the crystalline structure, and FT-IR was used to examine the surface. CuNWs showed XRD peaks at 43.3, 50.9, and 73.6° as characteristic 2θ peaks for crystallographic planes (111), (200), and (220), respectively. Purified CuNWs examined via FT-IR showed minimal IR absorbance, and minimally observable IR bands had magnitudes similar to the noise from atmospheric moisture.

### Synthesis of Silver Nanowires

Synthesis of silver nanowires followed a method previously described by Coskun et al.^61^ A typical synthesis started with 4.0 g of PVP and 5.0 mg of NaCl dissolved in 80.0 mL of EG and heated to 100 °C. In a separate glass beaker, 0.82 g of AgNO_3_ was dissolved in 40 mL of EG and heated to 120 °C in an oil bath. Once the two solutions are completely dissolved, the PVP and NaCl solution was combined with the AgNO_3_ solution. The combined mixture was equipped with a reflux condenser and a magnetic stirrer, and stirred at 160 °C for 1.5 h. After the initial 1.5 h, the temperature of the mixture was increased to 170 °C, and the mixture was stirred for 0.5 h. The mixture was cooled to room temperature and centrifuged at 21,300*g* to sediment the silver nanowires. The supernatant was disposed of, and 20.0 mL of ethanol was added to redisperse the residue. The centrifugation and redispersal steps were repeated three times to purify the silver nanowires. The final silver nanowire in ethanol dispersion was stored until further experiments.

Silver nanowires showed characteristic XRD 2θ peaks at 35.8, 44.9, and 64.9° for crystallographic planes (111), (200), and (220), respectively. There may be a smaller peak at 32.2°, which is associated with Ag_2_O on the surface of silver nanowires.

### Synthesis of Zinc Oxide Nanorods

The synthesis of ZnO nanorods followed procedures previously published by Hu et al.^62^ A typical synthesis started with dissolving 50.0 mg of ZnCl_2_, 5.0 g of Na_2_CO_3_, and 0.375 g of SDS in 22.5 mL of DI water. The mixture was then stirred for 30 min or until dissolved. The aqueous solution was transferred to a Teflon liner of a hydrothermal reactor and heated to 140 °C for 12 h. After the reaction, the mixture was cooled to room temperature and filtered using a paper filter. The solid product was washed three additional times using 100.0 mL of ethanol, followed by three times using 100.0 mL of DI water. Finally, the filtered material was dried in a circulating air oven at 60 °C for 4 h and stored.

ZnO nanorods showed characteristic XRD 2θ peaks at 32.1, 34.5, 36.4, 47.5, 56.7, 62.5, and 67.5° for crystallographic planes (100), (002), (101), (012), (110), (013), and (201), respectively.

### Electrospinning Polystyrene Fibers

Electrospinning of PS fibers followed the procedures published by Yeh et al.^63^ with some modifications. A typical electrospinning procedure began with the preparation of a polymer solution by dissolving 1.0 g of PS and 1.5 g of Triton X-100 into a mixture of 7.0 mL of DMF and 3.0 mL of chloroform. The mixture was heated to 60 °C under reflux and stirred for 8 h to fully dissolve. Once completely dissolved, the PS solution was loaded into a 3.0 mL syringe equipped with a 28 Ga flat-tipped stainless steel needle. A high-voltage source was attached to the stainless steel needle, and the needle was charged with a potential of 18 kV. At a rate of 0.40 mL/h, the PS solution was pumped through the needle and ejected toward a grounded stainless steel collector 15.0 cm away from the needle tip. A mat of PS fiber was collected on the grounded stainless steel collector, and the PS fiber mat can be recovered after the electrospinning process. The collected PS fiber mat was cut into 1.0 cm^2^ and dispersed into isopropanol for further experiments.

### Langmuir Monolayer Fiber Drawing of Various Microfibers

LMFD can be used to draw various 1D nanomaterials into microfibers, but CuNWs were used to demonstrate the process. A typical LMFD process started by dispersing CuNWs in hexane at a concentration of 10 mg/mL. To prepare the Langmuir monolayer trough, a Teflon basin with a cavity at least 5.0 cm long × 5.0 cm wide × 2.0 cm deep was cleaned using copious amounts of chloroform and acetone, followed by rinsing with DI water. The Teflon basin was filled to the brim with ethanol to form the Langmuir monolayer subphase. Approximately 5.0 mL of a dispersion of CuNWs was deposited onto the subphase in a dropwise manner, so the CuNWs floated at the air and ethanol interface without sinking into the subphase. By keeping the needle tip close to the subphase surface, the CuNW dispersion can be more successfully trapped on the surface. Barges closing at 10.0 mm/min can be used to reduce the surface area of the Langmuir monolayer and concentrate the 1D nanomaterials. Once CuNWs visibly cover the subphase surface, a thin microfiber can be pulled up using a stainless steel, 27 gauge, sharp-tipped, hypodermic needle. For more precise studies, a Wilhelmy plate can be used to monitor the surface pressure during the fiber-pulling process. The 27 gauge, stainless steel, sharp-tipped, hypodermic needle was used to puncture the Langmuir monolayer and lift the monolayer out from the subphase. A yarn-like microfiber of CuNW trailed behind the needle, and the microfiber can be collected for use in further studies. Similar procedures can be used to draw microfibers from other types of 1D nanomaterials.

### Characterization

The morphologies of nanomaterials were examined using a scanning electron microscope (SEM), JEOL 6330 TF. Crystal structure of nanomaterials’ X-ray diffraction (XRD) patterns were recorded by Bruker D8 Discover (Cu Kα λ = 1.54 Å). The surfaces of nanomaterials were examined using Fourier transform infrared spectroscopy (FT-IR) performed on a Nicolet iS50 FT-IR. A KSV NIMA KN 1002 was used to systematically alter the surface pressure during LMFD fiber drawing. Mechanical properties’ measurements were done using a Pro Test PT-1699 V tensile strength tester equipped with a 300 g load cell. Electrical conductivity was performed using a 4-point probe from KeithLink under ambient conditions. Electrical conductivity measurements were done immediately after preparing the CuNW microfiber. Fiji image processing software was used together with the OrientationJ plugin to evaluate the nanowires’ orientations in Figure 2.^54^ The OrientationJ functions used were the OrientationJ Analysis, OrientationJ Distribution, and OrientationJ Dominant Direction. Evaluation of orientation distribution used the finite-difference gradient.

## Data Availability

All data, figures, or codes used in this manuscript can be made available upon request.

## References

[1] Cho Y., Kim S., Cho H. J., Ham H., Lee K. E., Kang H. W., Jeong Y. W., Ahn J.-H., Choi N., Kang J. H. (2025). Cellular Nanointerface of Vertical Nanostructures: Impact of Size-Modulated
Nanopillar Arrays on Neuronal Morphology, Maturation, and Synapse
Formation. Small Struct..

[2] Johansson F., Jonsson M., Alm K., Kanje M. (2010). Cell guidance
by magnetic
nanowires. Exp. Cell Res..

[3] Yang F., Murugan R., Wang S., Ramakrishna S. (2005). Electrospinning
of nano/micro scale poly­(l-lactic acid) aligned fibers and their potential
in neural tissue engineering. Biomaterials.

[4] Duan X., Niu C., Sahi V., Chen J., Parce J. W., Empedocles S., Goldman J. L. (2003). High-performance thin-film transistors using semiconductor
nanowires and nanoribbons. Nature.

[5] Lv J., Zhou H., Ma X., Liu F., Peng L.-M., Qiu C. (2025). Self-Anchoring Process to Construct
Highly-Aligned-Carbon Nanotube
Transistors. ACS Nano.

[6] Park S., Pitner G., Giri G., Koo J. H., Park J., Kim K., Wang H., Sinclair R., Wong H.-S. P., Bao Z. (2015). Large-Area
Assembly of Densely Aligned Single-Walled Carbon Nanotubes Using Solution
Shearing and Their Application to Field-Effect Transistors. Adv. Mater..

[7] Fang Y., Sun M. (2015). Nanoplasmonic waveguides:
towards applications in integrated nanophotonic
circuits. Light: Sci. Appl..

[8] Law M., Sirbuly D. J., Johnson J. C., Goldberger J., Saykally R. J., Yang P. (2004). Nanoribbon Waveguides
for Subwavelength
Photonics Integration. Science.

[9] Takiguchi M., Fujii T., Sumikura H., Shinya A., Matsuo S., Notomi M. (2026). On-chip room-temperature CW lasing from a III–V
nanowire integrated with a Si photonic crystal platform. APL Photonics.

[10] Evers C. E., Vondrasek B., Jolowsky C. N., Park J. G., Czabaj M. W., Ku B. E., Thagard K. R., Odegard G. M., Liang Z. (2023). Scalable High
Tensile Modulus Composite Laminates Using Continuous Carbon Nanotube
Yarns for Aerospace Applications. ACS Appl.
Nano Mater..

[11] Luo J., Wen Y., Jia X., Lei X., Gao Z., Jian M., Xiao Z., Li L., Zhang J., Li T. (2023). Fabricating strong and tough aramid fibers by small addition of carbon
nanotubes. Nat. Commun..

[12] Broere L., Gogoi R., Barua A., Mathews N. G., Granroth S., Kolpakov K., Mohanty G., Peltola E., Sharma V. (2026). Durable copper
nanowires for flexible curvature sensors. Chem.
Eng. J. Adv..

[13] Peng Y., Liu H., Wu P., Zhang J. (2026). Electrically Conductive Inks as Programable
Material Systems for Adaptive, Multifunctional, and Sustainable Electronics. Adv. Eng. Mater..

[14] Won Y., Kim A., Yang W., Jeong S., Moon J. (2014). A highly stretchable,
helical copper nanowire conductor exhibiting a stretchability of 700%. NPG Asia Mater..

[15] Xu W.-H., Wang L., Guo Z., Chen X., Liu J., Huang X.-J. (2015). Copper Nanowires
as Nanoscale Interconnects: Their
Stability, Electrical Transport, and Mechanical Properties. ACS Nano.

[16] Barako M. T., Isaacson S. G., Lian F., Pop E., Dauskardt R. H., Goodson K. E., Tice J. (2017). Dense Vertically Aligned Copper Nanowire
Composites as High Performance Thermal Interface Materials. ACS Appl. Mater. Interfaces.

[17] Barako M. T., Roy-Panzer S., English T. S., Kodama T., Asheghi M., Kenny T. W., Goodson K. E. (2015). Thermal Conduction in Vertically
Aligned Copper Nanowire Arrays and Composites. ACS Appl. Mater. Interfaces.

[18] Peng W.-T., Chen F.-R., Lu M.-C. (2021). Thermal
conductivity and electrical
resistivity of single copper nanowires. Phys.
Chem. Chem. Phys..

[19] Im H.-G., Jung S.-H., Jin J., Lee D., Lee J., Lee D., Lee J.-Y., Kim I.-D., Bae B.-S. (2014). Flexible Transparent
Conducting Hybrid Film Using a Surface-Embedded Copper Nanowire Network:
A Highly Oxidation-Resistant Copper Nanowire Electrode for Flexible
Optoelectronics. ACS Nano.

[20] Kim J., Kim M., Jung H., Park J., Lee Y. (2023). Ultrastable 2D material-wrapped
copper nanowires for high-performance flexible and transparent energy
devices. Nano Energy.

[21] Xu X., Wang R., Nie P., Cheng Y., Lu X., Shi L., Sun J. (2017). Copper Nanowire-Based
Aerogel with Tunable Pore Structure
and Its Application as Flexible Pressure Sensor. ACS Appl. Mater. Interfaces.

[22] Zhao Y., Kang J., Huang W., Kong P., An D., He G. (2023). Copper Nanowire/Polydopamine-Modified
Sodium Alginate Composite Films
with Enhanced Long-Term Stability and Adhesion for Flexible Organic
Light-Emitting Diodes. ACS Appl. Mater. Interfaces.

[23] Zhong Z., Lee H., Kang D., Kwon S., Choi Y.-M., Kim I., Kim K.-Y., Lee Y., Woo K., Moon J. (2016). Continuous
Patterning of Copper Nanowire-Based Transparent Conducting Electrodes
for Use in Flexible Electronic Applications. ACS Nano.

[24] Chen Z., Rathmell A. R., Ye S., Wilson A. R., Wiley B. J. (2013). Optically
Transparent Water Oxidation Catalysts Based on Copper Nanowires. Angew. Chem., Int. Ed..

[25] Liu X., Sui Y., Yang X., Wei Y., Zou B. (2016). Cu Nanowires with Clean
Surfaces: Synthesis and Enhanced Electrocatalytic Activity. ACS Appl. Mater. Interfaces.

[26] Luo W., Guo Z., Ye L., Wu S., Jiang Y., Xu P., Wang H., Qian J., Zhou X., Tang H. (2024). Electrical-Driven Directed-Evolution
of Copper Nanowires Catalysts
for Efficient Nitrate Reduction to Ammonia. Small.

[27] Campos M. T., Pires L. S., Magalhães F. D., Oliveira M. J., Pinto A. M. (2025). Self-assembled
inorganic nanomaterials for biomedical applications. Nanoscale.

[28] Li Z., Fan Q., Yin Y. (2022). Colloidal
Self-Assembly Approaches to Smart Nanostructured
Materials. Chem. Rev..

[29] Ma R., Li S., Zhang S. (2025). Assembly and
Processing of One-Dimensional Subnanomaterials. Small.

[30] Cervantes-Sodi F., Vilatela J. J., Jiménez-Rodríguez J. A., Reyes-Gutiérrez L. G., Rosas-Meléndez S., Íñiguez-Rábago A., Ballesteros-Villarreal M., Palacios E., Reiband G., Terrones M. (2012). Carbon nanotube bundles
self-assembled in double helix microstructures. Carbon.

[31] Tilve-Martinez D., Lozano-Steinmetz F., Gómez-Palos I., J Vilatela J. (2025). Ordered silicon
nanowire bundle networks from aqueous dispersions. Nanotechnology.

[32] Chen Y., Liang T., Chen L., Chen Y., Yang B.-R., Luo Y., Liu G.-S. (2022). Self-assembly,
alignment, and patterning of metal nanowires. Nanoscale Horiz..

[33] Chai Z., Childress A., Busnaina A. A. (2022). Directed Assembly of Nanomaterials
for Making Nanoscale Devices and Structures: Mechanisms and Applications. ACS Nano.

[34] Huang Y., Duan X., Wei Q., Lieber C. M. (2001). Directed Assembly
of One-Dimensional Nanostructures into Functional Networks. Science.

[35] Zhang S., Pelligra C. I., Feng X., Osuji C. O. (2018). Directed Assembly
of Hybrid Nanomaterials and Nanocomposites. Adv. Mater..

[36] Reiser B., Gerstner D., Gonzalez-Garcia L., Maurer J. H. M., Kanelidis I., Kraus T. (2017). Spinning Hierarchical
Gold Nanowire Microfibers by Shear Alignment
and Intermolecular Self-Assembly. ACS Nano.

[37] Zhao L., Fan J., Gong C., Dyke A., Gao W., Li B. (2025). A Critical
Review on Recent Progress of Solution-Processed Monolayer Assembly
of Nanomaterials and Applications. Small.

[38] Bodik M., Maxian O., Hagara J., Nadazdy P., Jergel M., Majkova E., Siffalovic P. (2020). Langmuir–Scheaffer
Technique
as a Method for Controlled Alignment of 1D Materials. Langmuir.

[39] Yang P., Kim F. (2002). Langmuir–Blodgett
Assembly of One-Dimensional Nanostructures. ChemPhysChem.

[40] Hernández-Vélez M. (2006). Nanowires
and 1D arrays fabrication: An overview. Thin
Solid Films.

[41] Kimura Y., Cui Y., Suzuki T., Tanaka Y., Tanaka T., Toku Y., Ju Y. (2024). Growth of metal nanowire
forests controlled through stress fields
induced by grain gradients. Science.

[42] Su B., Wu Y., Jiang L. (2012). The art of aligning one-dimensional (1D) nanostructures. Chem. Soc. Rev..

[43] Foroughi J., Spinks G. M., Antiohos D., Mirabedini A., Gambhir S., Wallace G. G., Ghorbani S. R., Peleckis G., Kozlov M. E., Lima M. D., Baughman R. H. (2014). Highly
Conductive
Carbon Nanotube-Graphene Hybrid Yarn. Adv. Funct.
Mater..

[44] Nguyen A. N., Okamoto N., Abe R., Heriyanto A. D. M., Kumari N., Pado G., Tanimura S., Cho Y., Pandey M., Benten H., Nakamura M. (2024). Carbon Nanotube Yarns
Tailored Using Dispersants and Surfactants for Flexible and Wearable
Thermoelectric Generators. ACS Appl. Nano Mater..

[45] Zhong X.-H., Li Y.-L., Liu Y.-K., Qiao X.-H., Feng Y., Liang J., Jin J., Zhu L., Hou F., Li J.-Y. (2010). Continuous Multilayered Carbon Nanotube Yarns. Adv. Mater..

[46] Haider S., Al-Zeghayer Y., Ahmed Ali F. A., Haider A., Mahmood A., Al-Masry W. A., Imran M., Aijaz M. O. (2013). Highly aligned narrow
diameter chitosan electrospun nanofibers. J.
Polym. Res..

[47] Hosseinian H., Jimenez-Moreno M., Sher M., Rodriguez-Garcia A., Martinez-Chapa S. O., Hosseini S. (2023). An origami-based technique for simple,
effective and inexpensive fabrication of highly aligned far-field
electrospun fibers. Sci. Rep..

[48] Katta P., Alessandro M., Ramsier R. D., Chase G. G. (2004). Continuous Electrospinning
of Aligned Polymer Nanofibers onto a Wire Drum Collector. Nano Lett..

[49] Robinson A. J., Pérez-Nava A., Ali S. C., González-Campos J. B., Holloway J. L., Cosgriff-Hernandez E.
M. (2021). Comparative analysis
of fiber alignment methods in electrospinning. Matter.

[50] Peng J., Grayson M., Snyder G. J. (2021). What makes a material
bendable? A
thickness-dependent metric for bendability, malleability, ductility. Matter.

[51] Peng J., Snyder G. J. (2019). A figure of merit for flexibility. Science.

[52] Lin H., Zhang Z., Zhang H., Lin K.-T., Wen X., Liang Y., Fu Y., Lau A. K. T., Ma T., Qiu C.-W., Jia B. (2022). Engineering
van der Waals Materials
for Advanced Metaphotonics. Chem. Rev..

[53] Ma L., Wang Y., Liu Y. (2024). van der Waals Contact for Two-Dimensional
Transition Metal Dichalcogenides. Chem. Rev..

[54] Schindelin J., Arganda-Carreras I., Frise E., Kaynig V., Longair M., Pietzsch T., Preibisch S., Rueden C., Saalfeld S., Schmid B. (2012). Fiji: an open-source platform for biological-image
analysis. Nat. Methods.

[55] Oliveira O. N., Caseli L., Ariga K. (2022). The Past and the Future
of Langmuir and Langmuir–Blodgett Films. Chem. Rev..

[56] Wilhelmy L. (1863). Ueber die
Abhängigkeit der Capillaritäts-Constanten des Alkohols
von Substanz und Gestalt des benetzten festen Körpers. Ann. Phys..

[57] Harkins W. D., Boyd E. (1941). The States of Monolayers. J. Phys. Chem. A.

[58] Kaganer V. M., Möhwald H., Dutta P. (1999). Structure and phase transitions in
Langmuir monolayers. Rev. Mod. Phys..

[59] Li S., Chen Y., Huang L., Pan D. (2014). Large-Scale Synthesis
of Well-Dispersed Copper Nanowires in an Electric Pressure Cooker
and Their Application in Transparent and Conductive Networks. Inorg. Chem..

[60] Wang R., Ruan H. (2016). Synthesis of copper
nanowires and its application to flexible transparent
electrode. J. Alloys. Compd..

[61] Coskun S., Aksoy B., Unalan H. E. (2011). Polyol
Synthesis of Silver Nanowires:
An Extensive Parametric Study. Cryst. Growth
Des..

[62] Hu H., Huang X., Deng C., Chen X., Qian Y. (2007). Hydrothermal
synthesis of ZnO nanowires and nanobelts on a large scale. Mater. Chem. Phys..

[63] Yeh T., Yang P., Lin K., Zheng B.-W., Chen Y.-T., Chiou K. (2023). Transferrable Electrospinning
Nanofiber Meshes as Strongly Adhered
Scaffolds for Slippery Liquid-Infused Porous Surfaces. ACS Omega.

